# Young at Heart: Pioneering Approaches to Model Nonischaemic Cardiomyopathy with Induced Pluripotent Stem Cells

**DOI:** 10.1155/2016/4287158

**Published:** 2016-03-24

**Authors:** Aoife Gowran, Marco Rasponi, Roberta Visone, Patrizia Nigro, Gianluca L. Perrucci, Stefano Righetti, Marco Zanobini, Giulio Pompilio

**Affiliations:** ^1^Unit of Vascular Biology and Regenerative Medicine, Centro Cardiologico Monzino-IRCCS, Via Parea 4, 20138 Milan, Italy; ^2^Department of Electronics, Information and Bioengineering, Politecnico di Milano, Piazza Leonardo da Vinci 32, Building No. 21, 20133 Milan, Italy; ^3^Department of Clinical Sciences and Community Health, University of Milan, Via Festa del Perdono 7, 20122 Milan, Italy; ^4^Cardiology Unit, San Gerardo Hospital, Via Giambattista Pergolesi 33, 20052 Monza, Italy; ^5^Department of Cardiac Surgery, Centro Cardiologico Monzino-IRCCS, Via Parea 4, 20138 Milan, Italy

## Abstract

A mere 9 years have passed since the revolutionary report describing the derivation of induced pluripotent stem cells from human fibroblasts and the first in-patient translational use of cells obtained from these stem cells has already been achieved. From the perspectives of clinicians and researchers alike, the promise of induced pluripotent stem cells is alluring if somewhat beguiling. It is now evident that this technology is nascent and many areas for refinement have been identified and need to be considered before induced pluripotent stem cells can be routinely used to stratify, treat and cure patients, and to faithfully model diseases for drug screening purposes. This review specifically addresses the pioneering approaches to improve induced pluripotent stem cell based models of nonischaemic cardiomyopathy.

## 1. Introduction

The American Heart Association's Scientific Statement from the Council of Clinical Cardiology defines cardiomyopathy as “*a heterogeneous group of diseases of the myocardium associated with mechanical and/or electrical dysfunction, which usually (but not invariably) exhibit inappropriate ventricular hypertrophy or dilatation, due to a variety of etiologies that frequently are genetic*” [1]. Predominately, cardiac diseases involving the myocardium are caused by atherosclerosis and termed ischaemic cardiomyopathies. However, there is a plethora of diseases linked to the myocardium which are unconnected with atherosclerosis and not ischaemic in nature. Nonischaemic cardiomyopathies (NICM) are classified as primary and secondary, where the pathology in the former is confined to the heart whilst for the latter the cardiomyopathy is due to underlying systemic disease. The prevalence of NICM in patients enrolled in heart failure trials ranged from 19% to 53%; however, there may be significant bias in this data as other reports show much lower levels in unselected patient populations with greater gender balance [2]. The diagnosis of the specific NICM is not straightforward requiring careful clinical correlation, cardiac magnetic resonance imaging, and frequently myocardial biopsy [3]. Despite many NICM being genetic in origin, the exact underlying causes and pathological molecular mechanisms of NICM are not well characterised in comparison to ischaemic cardiomyopathy and many remain unknown [2]. Pathological tissue characteristics of the myocardium in NICM are inflammatory oedema, abnormal calcium handling, fatty infiltration, fibrosis, and the formation of scar tissue which has been shown to be the genesis of life threatening cardiac arrhythmia [1–3]. The causative factors associated with heart failure in NICM are numerous and heterogeneous in nature such as genetic mutations [1], excessive alcohol consumption, malnutrition, cytotoxic drugs, exposure to infectious agents, autoimmunity, and hyperthyroidism [2]. Logically the removal of the causative factor in NICM affects the clinical course of the heart failure; however, the causative factor is often difficult to eliminate or control, or is not known. Mortality rates (2-year follow-up) have been found to be better in NICM (48%; idiopathic dilated cardiomyopathy) compared to ischaemic cardiomyopathy (69%; coronary heart disease) [4]. Another study showed (5-year follow-up) that the survival rate was higher for NICM (69%) compared to ischaemic cardiomyopathy (59%) [5]. Responses of NICM patients to essential cardiac drugs (e.g., angiotensin-converting enzyme inhibitors, beta-blockers, and diuretics) do not differ to that of patients with ischaemic cardiomyopathy [6]. However, 2 trials (Prospective Randomised Study of Ventricular Function and Efficacy of Digoxin, PROVED [7], and Randomised Assessment of Digoxin and Inhibitors of Angiotensin-Converting Enzyme, RADIANCE [8]) have shown positive differences (compared to ischaemic heart disease patients) in left ventricle ejection fractions in patients with nonischaemic dilated cardiomyopathy (DCM) receiving digoxin alone or in combination with inhibitors of angiotensin-converting enzyme. Furthermore, other drugs such as pentoxifylline (an inhibitor of tumor necrosis factor-alpha), growth hormone, and amiodarone (a class III antiarrhythmic agent) were found to elicit better responses in NICM patients [6].

In spite of this, treatment efficacy becomes limited for NICM patients with gravely decreased cardiac functions which can only be resolved by heart transplant. Alternatively, cardiac stem cell therapy is a developing approach which is currently undergoing clinical trials to assess the ability of various stem cells to functionally rejuvenate the heart [9, 10]. Numerous appraisals of stem cell transplantation for cardiac diseases have concluded that although safe, cell transplantation does not promote significant gains in cardiac functions and therefore further investigations are needed in forthcoming trials [11–13]. Thus, enhancing cardiac cell therapy for myocardial regeneration requires the development of pioneering approaches stemming from multiple disciplines: biology, engineering, and clinical medicine.

Pluripotent stem cells (PSCs) derived from early embryos (embryonic stem cells (ESCs) [14]) or induced following the reprogramming of somatic cells (induced pluripotent stem cells (iPSCs) [15]) represent a groundbreaking advance in regenerative medicine. Whilst both types of PSCs are indistinguishable in terms of pluripotency, iPSCs have a distinct advantage over ESCs as they can be autologously generated and are not connected to major ethical concerns. Cells derived from both ESCs and more recently iPSCs have been used in clinical trials tackling tissue regeneration and as drug screening tools. Furthermore, iPSCs can be derived from individuals with a genetic disease (or carriers) and thus are an important unlimited cell source which can be differentiated into specific cell types, including cardiomyocytes (iPSC-CMs) that display the relevant pathophysiology. However, continued innovations are needed to maximise the proposed power of stem cell derived cardiomyocytes as cell products and laboratory tools.

Significant advances in generating stem cell derived human cardiomyocytes and bioengineering strategies offer an unprecedented opportunity to create bioengineered systems for the more accurate simulation of the native environment. Considerable progress has been made on the use of static 2D cultured human stem cell derived CMs [16–20]. However, the predictability of such systems is still limited. Microtechnologies have been increasingly adopted to study PSCs and progeny cells* in vitro*, particularly to build 3D culture systems, which are beginning to replace existing traditional platforms. The rapid fabrication of polydimethylsiloxane- (PDMS-) based biocompatible microfluidic devices, especially suitable in bioengineering, was made possible by the widespread use of soft lithography techniques developed during the 1990s by the Whitesides group [21]. Besides the intrinsic advantages of miniaturisation, such as the reduction of reagents and cell numbers required for each experiment, microdevices allow for the accurate generation of 3D tissues and a high control over the cell microenvironment providing well-defined chemical, geometrical, mechanical, or electrical cues [22, 23]. Moreover, the integration of microelectrodes allows the stimulation of heart cells in parallel with monitoring their contraction. Particular effort has been recently spent on the development of heart-on-chip platforms by taking advantage of microfabrication, biomaterials, and iPSCs [24, 25]. These systems represent* in vitro* models capable of imitating the natural heart muscle and promise to increase the predictability of drug screening and disease modelling at unprecedented levels.

## 2. Induced Pluripotency

The discovery of iPSC technology has revolutionised stem cell based research and personalised medicine. This technology permits the reprogramming of terminally differentiated somatic cells and reprogram them into intermediary iPSCs by the ectopic expression of pluripotency factors (octamer-binding transcription factor 4 (OCT4) and SRY- (sex determining region Y-) box 2 (SOX2), kruppel-like factor 4 (Klf4), and V-Myc avian myelocytomatosis viral oncogene homolog (c-Myc); OSKM) [26]; the later addition of Nanog and Lin28 enhanced efficiency [27]). Upon overexpression of these factors, somatic cells are capable of activating endogenous core-pluripotency pathways which modulate the expression of genes regulating cell fate [28]. The ensuing iPSCs can be differentiated into functional cells of interest, for example, neurons [29], cardiomyocytes [30], vascular cells [31, 32], chondrocytes [33], pancreatic cells [34], and hepatocytes [35], amongst other cell types resident in organs originating from all 3 germ layers. Since the initial report of the iPSC platform, there have been a series of methodological evolutions which have greatly increased the efficiency of the process and the appeal for translational use.

Induced pluripotency has been shown to be a universally applicable method [36] as iPSCs have been derived from a number of founder cells other than skin fibroblasts, such as renal epithelial cells [37], endometrial stromal cells [38], mesenchymal stem cells [39], differentiated T-cells [40], and peripheral blood mononuclear cells (PBMCs) [41]. Despite the broad range of founder cells, some types, for instance, PBMCs, are more applicable for the derivation of iPSCs than others. Some reasons for this enhanced suitability are the less invasive nature of sampling needed to obtain the cells, the preexistence of “in-house” sample processing logistics, the ease of* in vitro* expansion and purification steps, the low exposure to environmental mutagens, and reduced processing times.

The mode of delivering reprogramming factors and the selection of reprogramming factors themselves have been subject to major advances. The field has moved away from retro- [15, 26] and lentiviral [27] mediated transfection to using nonintegrating viral vector-based methods that preserve high reprogramming efficiency rates whilst maintaining genome integrity (e.g., Sendai virus) [42]. Furthermore, Sendai virus transduced cells do not need further transgene excision and the presence of virus in the cell cytosol can be cleared by elevated temperatures or siRNA treatment. In spite of this, the original retro- and lentiviral based systems are still routinely utilised for basic research and disease modelling purposes as there is less onus on translational potential. Groups with a translational outlook favour nonintegrating methods of reprogramming such as episomal vectors [41], microRNAs [43], and protein transduction [44, 45]. These clinically orientated approaches to reprogramming come at the cost of reduced efficiency. However, when combined with the original OSKM reprogramming factors, nonintegrating pluripotency factors such as miRNA, small molecules, and epigenetic regulators have drastically increased reprogramming efficiency rates from 0.01% (OSKM) [36] to a reported near 100% efficient method termed deterministic reprogramming [46]. In an effort to decode and control the molecular mechanisms of induced pluripotency, researchers have suggested that there is a more nuanced and fluid control of pluripotency [47, 48] which has been termed the “seesaw model” entailing the balance between endogenous/exogenous pluripotency and lineage-specifying factors [49]. Indeed, investigations have shown that many of the core-pluripotency factors OCT4 and SOX2 can be replaced by lineage-specifying genes (e.g., GATA binding protein 3 (GATA3), zinc finger protein 521 (ZNF521), orthodenticle homeobox 2 (OTX2), and paired box 6 (PAX6)) during human iPSC generation [50]. Further studies, based on preexisting knowledge regarding the induction of pluripotency in the absence of exogenous genes [51, 52], have demonstrated that small molecules can replace all the core-pluripotency genes (including OCT4) to induce the fully chemical-based reprogramming of murine fibroblasts [53], albeit at very low efficiency rates.

Lastly, protocols for culturing iPSCs with fully defined* xeno*-free media and matrices have evolved in parallel to advances in efficient reprogramming methods. Several commercially available work flow products now permit the fully defined isolation and culture of human iPSCs without the need for specialist knowledge or equipment. These products have vastly improved the efficiency and quality and certainly have reduced the labor costs (in terms of work hours) associated with performing iPSC-based research. Remarkably, the entire process of deriving, characterising, and differentiating multiple iPSC lines in parallel has now been automated in a modular, robotic high-throughput platform [54]. The advances in streamlining, upscaling, and automation suggest that iPSCs will soon become useful in large-scale efforts to tackle complex diseases, stratify patient treatment responses, and adopt personalised drug regimes. Furthermore, the creation of national human leukocyte antigen (HLA) haplotype-matched iPSC biobanks large enough to cover entire populations may serve to cover medical situations where there is an acute need for replacement cells, for example, stroke and myocardial infarction [55–57].

From these richly diverse procedures to obtain iPSCs, scientists and clinicians are free to tailor their choice of methodological approach to generate iPSCs based on their own priorities be it transduction efficiency, high reprogramming fidelity, authentic disease modelling, or clinical use.

## 3. iPSC Differentiation and Derivation of Cardiomyocytes

The most common destiny of iPSCs is to undergo directed differentiation where the subsequent differentiated cells can be used for a myriad of purposes such as disease models, drug toxicity screens, novel drug discovery, tissue developmental models, and cell-therapy products. Many of these applications require large numbers of cells (in the order of 10^7^ to 10^9^ cells) generated in a chemically defined,* xeno*-free, reproducible, scalable, and cost-effective manner [58, 59].

Current CM differentiation protocols, although now efficient compared to early protocols (e.g., fetal bovine serum-based embryoid body [30] or inductive coculture on visceral endoderm-like (END-2) cells [60]), still lack the rigorous derivation standards mentioned above: for example, many still involve media containing animal-based components [61]. Most CM differentiation protocols rely on emulating embryonic mesoderm induction signals such as bone morphogenetic protein (BMP), Notch, and Wnt signalling [62], with the following generation of heart-field-specific progenitors by inhibiting Wnt, BMP, and TGF-beta signalling [58, 61]. CM yields of 10^6^–10^9^ cells with high purity rates (80%) have been reported, while higher purity (95–99%) levels are achievable following metabolic selection by glucose deprivation [58, 61, 63]. From a translation point of view, metabolic CM selection is preferable to genetic selection strategies [64] as the former does not involve genetic modification. However, despite high levels of CM purity more in-depth characterisations of resulting CMs have revealed that there is a mixture of CM subtypes (atrial-, ventricular-, and nodal-like CMs) in the postdifferentiation cell population [30, 59]. This issue is of paramount importance since there is growing demand for CM subtype-specific cell-therapy products suitable for myocardial repair. Interestingly, augmenting retinoic acid signalling with a pan-retinoic acid receptor antagonist BMS-189453 elevates the number of ventricular CMs (as assessed by the presence of ventricular-specific markers IRX-4 and MLC-2V, ventricular-like action potentials, and calcium handling) [65], indicating that this signalling pathway could be of use in controlling CM subtype specification. In order to more accurately reflect the cellular composition of the myocardium, the complexity of iPSC-derived cardiac tissue needs to be expanded, for example, including a wider range of cardiovascular cells and more mature CMs [66].

Alternatively, direct cardiac reprogramming* in vitro* offers the potential to skip the intermediary iPSC stage in the production of CMs for heart regeneration [67]. A number of groups have demonstrated that induced CMs (iCMs) can be generated from human fibroblasts reprogrammed with cardiac-specific transcription factors (GATA binding protein 4 (GATA4), heart and neural crest derivatives expressed 2 (Hand2), T-box transcription factor 5 (T-box5), myocardin, myocyte enhancer factor 2C (Mef2c), estrogen-related receptor-beta (Esrrb), and mesoderm posterior basic helix-loop-helix transcription factor 1 (Mesp1)), and muscle-specific microRNAs (miR-1 and miR-133) [68–70]. However, these approaches have not been without problems as reprogramming efficiencies are low (0.5–20%) and reproducibility has been problematic [71, 72]. In contrast, the direct reprogramming of murine cardiac fibroblasts* in vivo* is more efficient which raises the tempting possibility that the direct delivery of cardiac reprogramming factors into the damaged heart may be possible [36]. Nevertheless, the matters of potential genomic damage caused by the random integration of transcription factors by retroviral vectors, the risk of arrhythmia, the presence of mixed CM subtypes with varying degrees of maturity, and the lack of translation into large animal or human experiments remain major hurdles for this emerging avenue of iPSC-based cardiac cell therapy. Furthermore, the length of time that iCMs survive posttreatment and indeed whether this approach is more suitable for acute or chronic cardiac clinical-needs remain open questions. To overcome these challenges, a deeper understanding of the mechanisms governing cell reprogramming is essential which will be aided by transcriptomic [73] and epigenomic analyses [36].

## 4. Harnessing the Functional Maturity of CMs Derived from iPSCs

One of the most pervasive obstacles in iPSC research and development is the generation of mature iPSC-CMs and the full recapitulation of disease. iPSC-CMs express high levels of cardiac genes and display spontaneous beating (48.6 average beats per minute), which upon electrophysiological analysis can be allotted to predominantly ventricular-like (48–74%) but also atrial- and nodal-like action potentials (APs) [30, 74–77]. However, upon closer examination it has been shown that iPSC-CMs do not exhibit many morphological and functional characteristics of adult CMs. For instance, iPSC-CMs are small in size with multiangular symmetries [78], have unorganised sarcomeres and altered Ca^2+^ transients [79] due to the lack of t-tubuli [80]. iPSC-CMs display altered APs resulting from discrepancies in certain cardiac ion channels [75, 81], namely, low inward rectifier potassium (Kir) and high cardiac pacemaker “funny” (I*f*) channel densities [75]. Thus, iPSC-CMs are electrophysiologically immature and have a depolarised membrane potential and slow AP upstroke velocities (reviewed in [81]). Clearly these differences impact the usefulness of iPSC-CMs as drug screening and disease modelling tools [82] and represent a serious safety issue in relation to the development of cell-therapy products. This coupled to the increasing demand for novel cardiovascular therapies and tailored patient treatment plans signifies that improving iPSC-CMs is a clinical imperative [83].

Long term culture, electrical stimulation, and exposure to mechanical forces are some of the pioneering strategies to influence the maturity of iPSC-CMs in order to more faithfully reprise the* in vivo* phenotype. Additionally, recreating the* in vivo* tissue- and cytoarchitecture by culturing iPSC-CMs in 3D scaffolds in the presence of other cardiovascular cell types can also influence the maturity of iPSC-CMs so that they may become more attractive for future use in multiple applications [82, 84]. It is conceivable that the maturity of iPSC-CMs maybe tunable to offer the best possible tool to yield relevant and useful information which is balanced with the degree of complexity in the engineered model. Reproducing a myocardial-like environment* in vitro*, through biochemical (e.g., natural extracellular matrix-like growth substrate, soluble factors), mechanical (e.g., cyclic strain), and electrical stimulation, has been reported to be a successful strategy to induce cell differentiation into mature CMs [85]. Likewise, in order to generate a more functional and physiological-like tissue, different strategies have been exploited to guide cells to form 3D constructs by promoting the spontaneous formation of the tissue without the use of exogenous synthetic materials or driving the autoassembly of cell laden hydrogels at specific anchoring points [24]. Cells embedded in hydrogels (i.e., collagen I, fibrin, and Matrigel*™*) exhibited the ability to organise themselves around fixed or mobile posts [86–88] or hydrogels could be inserted into casting moulds to form circular strips circumferentially aligned around cylindrical structures [89, 90]. Once the 3D constructs are formed, the posts can be used as passive components to evaluate cardiac contractile activity by optically monitoring postdeflection in response to forces generated by constructs [91] either spontaneously or under the effect of electrical pacing. Furthermore, posts have also been exploited as active components (i.e., springs) to control tissue remodelling and stiffening. For example, modulating the elastic modulus of posts or adhering nickel spheres on the post tops so that their position can be controlled through magnetic field imposition has been shown to control tissue stiffening during tissue remodelling [92]. Moreover, mechanical stimulation enhanced a physiological hypertrophy and architecture of cardiac constructs as demonstrated by Tulloch and colleagues [93]. Specifically, the imposition of uniaxial cyclic stress to iPSC-CMs embedded in a 3D collagen matrix resulted in cardiac constructs showing fiber alignment and myofibrillogenesis and sarcomeric banding [93] and increased the duration of cellular action potentials. Additionally, although mechanical stimulation was able to guide the maturation of CM calcium handling machinery [94], it was not able to achieve a CM maturation stage similar to that obtained with electrical stimulation [95]. Recent investigations based on exploiting the spontaneous engineered heart tissue (EHT) formation techniques discussed above have demonstrated that coupling mechanical and electrical stimulations supports a better functional maturity of cardiac tissue. Indeed, Miklas et al. [96] obtained cardiac microtissues exhibiting more relevant contraction amplitudes and forces with a superior development of sarcomeric structures by applying electromechanical stimulation (1 ms biphasic 3-4 V/cm pulses at 1 Hz; 5% constant stretch) to neonatal rat CMs seeded in collagen gels. These results are in agreement with Boudou and coworkers' demonstration that the combination of electrical stimulation (1 ms biphasic 6 V/cm pulses at 0.2 Hz) and auxotonic load strongly improves both the structure and function of cardiac microtissues [22]. Godier-Furnémont et al. [97] recently formed EHT strips using a mixture of collagen I, Matrigel, and neonatal rat heart cells, proving that electromechanical stimulation performed at physiological frequencies supports EHT functional maturation. Furthermore, precondensed EHT strips subjected to dynamic mechanical stretch (supporting auxotonic contraction) in combination with electrical stimulation (3 ms biphasic 5 V pulses at 4 Hz) showed a positive force-frequency relationship (FFR) and cells within the construct exhibited mature sarcoplasmic calcium handling machinery [97].

CMs typically act in concert with fibroblasts, vascular cells, and neurons, and these cell-cell interactions tightly regulate heart development and function* in vivo* [98]. Coculture systems reproducing this cell heterogeneity have been reported to enhance cardiac tissue performance and functionality* in vitro*. Cardiac fibroblasts (CFs), in physiological conditions, provide a scaffolding structure for CMs and coordinate the pump function of the heart [99].* In vitro* 3D cocultures of CFs with CMs in physiological ratios (2 : 1) conferred enhanced functionalities on engineered cardiac microtissues, improving cellular elongation, alignment, viability, and cell-cell interaction via connexin and cadherin proteins [100–102]. Sympathetic neurons regulate heart rate, excitation conduction velocity, and myocardial contraction/relaxation mechanisms. Takeuchi et al. developed different systems [103–105] employing multielectrode array substrates to compartmentally coculture autonomic nervous system neurons with CMs or iPSC-CMs, achieving the formation of functional synapsis between the two cell populations. This method allowed the control and modulation of the cardiac contraction rhythm in a* quasi*-natural way, by electrically stimulating sympathetic neurons [106]. In the native myocardium, there is a high density of capillaries for cardiac cells, with a proportion of about three endothelial cells (ECs) per CM [107]. Integrating a vascular network within* in vitro* cardiac constructs enhanced CM survival and spatial reorganisation in a 3D configuration [108] and can thus increase the physiological relevance of generated tissues for fundamental biological studies or for implantation in patients. In the last years, many devices have been designed to guide perfusable microvascular network formation in a highly organised way by shaping luminal structures in the substrates to receive and arrange ECs. Exploiting this technique, Vollert et al. [109] increased CM density within contracting EHTs, generating small perfusable tubular structures. The Radisic group engineered a cardiac tissue around an organised capillary network grown between a vein and an artery, which improved construct functional properties, cell striation, and cell-cell junctions [110].

Table 1 lists a set of microdevices, together with the particular stimulating features, developed to differentiate* in vitro* stem cells toward the cardiac lineage.

## 5. Modelling NICM Using iPSCs

**Figure 1:**
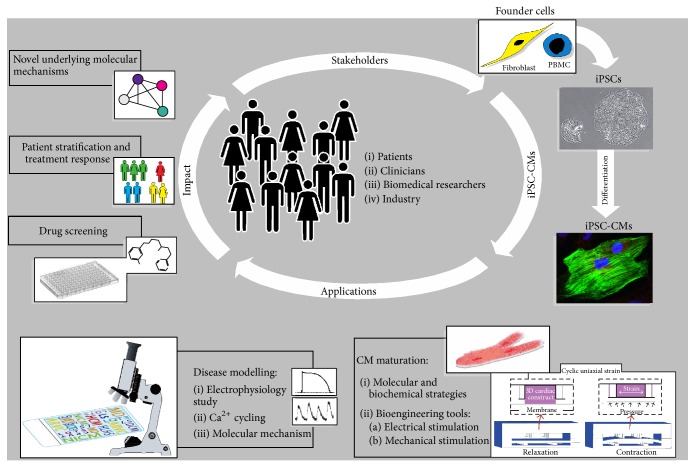
Modelling cardiac diseases with iPSC-CMs. Numerous stakeholders are involved in modelling cardiac diseases using iPSC-CMs: patients, patients' family members, or anonymous biobank donors who provide the founder cells, for example, skin fibroblasts or PBMCs, and also clinicians, biomedical researchers, and members of industry who participate in the collection of samples, the generation of iPSCs followed by differentiation into iPSC-CMs, and modelling underlying cardiac pathology and using state-of-the-art approaches, for example, bioengineered stimulating microdevice [25] and drug screening, to advance the impact of iPSC-CMs.

Since there is a low probability of obtaining cardiac muscle biopsies and with the high risk involved in these procedures, cardiovascular disease models using animals or animal-derived tissues/cells are the major ways to identify pathophysiological mechanisms and screen for drug toxicity. However, systematic reviews have shown that translating animal model findings into a human setting is hampered by the failure of animal models to fully recapitulate human disease [116, 117] further exacerbating the lack of new cardiovascular drugs [83] and the high failure rates in drug development due to cardiotoxicity [118, 119]. This emphasises the need for alternative approaches. Hence, iPSC-CMs represent a fruitful avenue to fill this gap and judging from the flurry of publications involving iPSC-CMs, which followed the initial description of iPSC technology, one would assume that the application of iPSC-CMs to model cardiac diseases has been extremely successful. To an extent this is true; however, so far the cardiac diseases modelled using iPSC-CMs are confined mostly to early onset monogenic diseases and thus are relatively easy to model with iPSC-CMs which resemble immature fetal CMs. A more daunting task is the replication of adult onset cardiac diseases associated with multiple genes, cardiac diseases with unknown pathological mechanisms, or subclinical symptoms. Yet the move towards this era is evident in the evolving literature on cardiac disease modelling with evermore complex cardiac diseases being demonstrated in iPSC-CMs. Indeed, several new insights have been gleamed following the recapitulation of cardiac diseases in patient specific CMs such as new disease mechanisms and responses to potential novel therapeutics.  Figure 1 illustrates the application of iPSCs in cardiovascular research.

Dilated cardiomyopathy (DCM), the most common form of NICM (Table 2 lists NICMs modelled with patient iPSC-CMs), is caused by mutations in cardiac troponin T (cTnT) gene and was successfully recapitulated in CMs induced from patient specific iPSCs (DCM-CMs) by Sun et al. [120]. DCM-CMs displayed abnormal punctate distribution of sarcomeric *α*-actinin and blockade of the beta-adrenergic pathway with metoprolol enhanced myofilament organisation and conferred resistance to mechanical damage. Furthermore, partial functional improvement and increased [Ca^2+^]_*i*_ were observed when sarcoplasmic/endoplasmic reticulum calcium ATPase 3 (Serca2a) was overexpressed in DCM-CMs. Another iPSC-CM-modelled cardiac disease associated with mutations in genes encoding sarcomeric proteins is hypertrophic cardiomyopathy (HCM) which is responsible for arrhythmias and sudden cardiac death [121]. HCM-CMs containing mutations in the myosin heavy chain 7 (MYH7) gene were pathologically enlarged and had contractile arrhythmias with dysregulated Ca^2+^ cycling and concentrations which responded favourably to verapamil (a L-type Ca^2+^ channel blocker). Additionally, HCM-CMs showed increased levels of hypertrophic-related genes (GATA4, cTnT, MYH7, and myosin light chain 2 (MYL2)) which was evident following prolonged culture (40 days).

Arrhythmogenic right ventricular cardiomyopathy (ARVC), also known as arrhythmogenic cardiomyopathy, is a cardiac disease of genetic origin which occurs due to mutations in cardiac desmosomal proteins, for example, plakophilin-2 (PKP2), which leads to ventricular tachyarrhythmias progressing to heart failure. ARVC has been modelled with patient iPSC-CMs by several groups [122–124]. ARVC-CMs displayed reduced PKP2, connexin-43 (Cx43), and peroxisome proliferator-activated receptor- (PPAR-) gamma expression, increased lipogenesis and enhanced adipogenic differentiation potential, and other electrophysiological irregularities (e.g., prolonged field potential risetime). Intriguingly, a model system developed by the Chen group [124, 140] showed that, in order to fully recapitulate meaningful ARVC disease readouts, patient specific iPSC-CMs needed to be metabolically matured and stimulated with activators of the PPAR-gamma pathway which is hyperactivated in ARVC right ventricles [125]. This approach highlights the need to carefully consider the maturation state of iPSC-CMs and the culture conditions before constructing disease readouts which may, at worst, be merely artificial features of a growth environment with insufficient recapitulation of the disease pathophysiology.

Genetic ion channelopathies such as long QT syndromes (LQTS) have been adequately modelled by different groups: LQT1 by Moretti et al. [74] and LQT2 by Lahti et al. [76], Matsa et al. [126], and Spencer et al. [127]. These models demonstrated several disease hallmarks in CMs derived from symptomatic patients' iPSCs, for example, prolonged AP duration with lower peak amplitudes, abnormal colocalisation of potassium channel voltage gated KQT-like subfamily Q member 1 (KCNQ1) to the endoplasmic reticulum (ER), decreased and altered delayed rectifier K^+^ current S (*I*_Ks_) currents, early after depolarisations (EADs) upon adrenergic challenge, and ameliorating responses to antiarrhythmic drugs. Additionally, Brugada syndrome or LQT3 has also been modelled with patient specific iPSC-CMs by two groups [128, 129] where again altered electrophysiological profiles such as reduced upstroke velocity, decreased inward Na^+^ channel (*l*_Na^+^_) density, and longer action potential duration (ADP90) with quicker pace frequencies were evident. Interestingly, the cardiac pathology of Timothy syndrome, which is a more complicated LQTS as it involves defects in both K^+^/Na^+^ channels, was demonstrated by Yazawa et al. [130]. Disease-specific electrical defects were shown in Timothy syndrome patients' iPSC-CMs such as low beating rate (bradycardia) and lower inactivation of L-type Ca^2+^ channels which were reversed by roscovitine, a cyclin-dependent kinase inhibitor that increases voltage-dependent inactivation. Lastly, catecholaminergic polymorphic ventricular tachycardia (CPVT), a stress-induced ventricular rhythm disease caused by mutations in the cardiac ryanodine receptor isoform 2 (RyR2) gene, has also been shown in CMs derived from iPSCs isolated from patients with CPVT (CPVT1-CMs) [131, 132]. CPVT1-CMs developed arrhythmias, delayed after depolarisations, abnormal Ca^2+^ handling, and negative chronotropic responses following isoproterenol treatment.

Carvajal-Vergara et al. [133] have generated CMs from patients with LEOPARD syndrome (LS) which causes hypertrophic cardiomyopathy due to mutations in the protein tyrosine phosphatase nonreceptor type 11 (PTPN11) gene encoding the protein tyrosine phosphatase containing a Src homology 2 domain (SHP2) phosphatase protein which is involved in rat sarcoma mitogen-activated protein kinase (RAS-MAPK) signalling. LS-CMs had increased median surface area and sarcomere assembly with a dephosphorylated calcineurin-dependent delocalisation of nuclear factor of activated T-cells, cytoplasmic calcineurin-dependent 4 (NFATc4) to the nuclei, and abnormal RAS-MAK signalling (epidermal growth factor receptor (EGFR) and mitogen-activated protein kinase kinase (MEK1) phosphorylation). Captivatingly, the alterations in RAS-MAK signalling were observed in iPSCs not iPSC-CMs indicating that the disease pathological mechanisms become altered as early as the cells of the inner cell mass in the developing embryo (the natural equivalents of iPSCs). Furthermore, iPSC-CMs from patients with hypoplastic left heart syndrome (HLHS) have been used to model the disease mechanisms of HLHS [134] which to date remain unclear. Cardiovascular malformations and cardiomyopathy are present in the early HLHS foetus. HLHS-CMs displayed downregulated mesoderm posterior basic helix-loop-helix transcription factor 1 (MESP1) and cTnT and randomly arranged myofibrils, RyR2 dysfunction, and lower beating rates. Coupled to these differences, changes in HLHS-iPSCs were also noted, specifically in cardiogenic capability of diseased iPSCs, indicating that both developmental and functional failings in iPSCs and CMs, respectively, may be responsible for impaired cardiogenesis in HLHS.

Dystrophinopathies are a group of X-linked genetic muscle diseases that are caused by mutations in the dystrophin gene which encodes the dystrophin protein. Muscular dystrophy (MD) patients have varying disease phenotypes dependent upon the severity of the mutation [141]. Although skeletal muscle is the main tissue affected in dystrophinopathies, MD patients are increasingly dying from cardiac disease typified by cardiomyopathy complicated by arrhythmias [135]. In late 2013 and early 2014 a trio of papers [136–138] presented the derivation of iPSC-CMs from Duchenne MD patients (DMD-CMs) and the successful demonstration of CM-specific pathology associated with this early onset severe form of MD. In 2015 an additional report of DMD-CMs followed on from the initial studies, this time also focusing on the Duchenne type of MD [139]. Interestingly, certain groups investigating Duchenne MD aimed to restore dystrophin expression through various approaches: exon-skipping antisense oligonucleotides [142], dystrophin minigenes [136], human artificial chromosome containing dystrophin sequences [137], and genome editing with TALENs or CRISPR-Cas9 [143]. Notably, the Elvassore group [137] used a growth substrate with a relevant physiological stiffness (15 kPascal) which improved the maturity of DMD-CMs which displayed mature sarcomeric organisation and shorter calcium transients (<1 s). The reports by Guan et al. [138] and Lin et al. [139] focused on the molecular mechanisms of MD cardiac disease. Specifically these groups found that the lack of dystrophin in DMD-CMs resulted in elevated Ca^2+^ levels and abnormal Ca^2+^ handling, mitochondrial permeability pore opening, increased susceptibility to hypotonic stress and elevated cardiac injury markers (creatine kinase-MB and cardiac troponin I) [138], and increased apoptosis [139]. Some of these molecular mechanisms were reversed in response to treatment of DMD-CMs with different potential MD therapeutics such as the membrane sealant Poloxamer 188 [139]. Additionally, Guan et al. [138] provided evidence that MD heart failure develops largely through independent mechanisms not linked to respiratory failure which represents a groundbreaking advance in the understanding of MD cardiac disease.

Undoubtedly, iPSC-CMs are a promising tool for cardiac disease modelling despite their functional immaturity. However, avoiding artifice is essential when interpreting results generated from iPSC-CMs. Thus, focusing on a palpable defect present and identifiable in patients' CMs which corresponds to a clinically meaningful component of the underlying disease and redressing the lack of pathophysiological cues in the cell-culture environment are the leading avenues of research which should generate more powerful disease models.

## 6. Value to the Clinic and Future Perspectives

Several national and international efforts to build iPSC biobanks point towards an optimistic future for the clinical application of iPSC-CMs, even in situations where the need is acute, for example, myocardial infarction. Biobanks aim to accumulate stocks of iPSCs which have been prepared under GMP conditions and have a fully traceable derivation and characterisation record. Furthermore, iPSC biobanks will curate samples from individuals with genetic disorders and produce other iPSC lines from healthy individuals for use in HLA-haplotype-matched allografts. These steps will help the clinical translation of basic research on cells derived from iPSCs and the development of iPSC lines for use in drug toxicology screening. Unfortunately, the lack of standard operating procedures for the culture of iPSCs, cell heterogeneity in iPSC cultures and progeny cells, genomic instability, and potential tumourigenicity implies that significant hurdles remain [144] and further basic research is needed in parallel to meticulously planned safety-focused clinical trials. Intriguingly there is an apparent shift towards the use of allogeneic rather than autologous iPSCs as evident by the suspension of the world's first clinical trial of iPSCs (for age-related macular degeneration), other clinical trials altering trial protocols to utilise allogeneic over autologous iPSCs and perhaps most tellingly the advent of iPSC biobanks stocking HLA-haplotype diverse iPSCs. Notably it has been estimated that an iPSC biobank containing between 50 and 200 lines of HLA-haplotype characterised iPSCs would adequately provide matched cells for millions of individuals [56, 57]. Thus, allograft cells could be more advantageous over allogeneic cells.

Cardiovascular trials of iPSC-based therapies are likely imminent and it will be interesting to learn the details regarding the source of iPSCs (autologous or allogeneic), maturity stage of the transplanted cells, and whether any bioengineering approaches were used in the cell product, for example, growth of cells on scaffolds. Overall, this is a period of immense opportunity to refine protocols and discover the latent potential of iPSCs in the cardiology clinical setting.

## Figures and Tables

**Table 1:** Cardiac differentiation in microdevices featuring high control over physicochemical stimulation.

Physics	Stimulation type	Cell environment	Cell type	Readout	Reference
Biochemical	Matrigel, B27 minus insulin supplement	3D	hiPSCs	Sarcomeric *α*-actin, and DAPI	Mathur et al. (2015) [24]

Biomimicry	Nanofiber scaffolds (macroscale)	3D	Embryoid bodies (EBs) from mESC	Sarcomeric *α*-actin, and connexin 43	Ghasemi-Mobarakeha et al. (2013) [111]
	Collagen type I interspersed with laminin	3D	EBs from mESC	PECAM (FITC), beating cells	Battista et al. (2005) [112]

Electrical	Electrodes – 304 stainless steel and titanium rods (10 cm × 1.3 mm)	2D	hESC	Troponin T, beating cells	Serena et al. (2009) [113]
	Indium tin oxide electrodes	2D	hASC	Connexin 43, F-actin, and beating cells	Tandon et al. (2010) [114]
	Carbon electrodes	3D	mPSC	Contraction force measurement	Boudou et al. (2012) [22]
	Electrodes in a Petri dish after biowire maturation	3D	hPSC	Sarcomeric *α*-actinin, Troponin T, and beating cells	Nunes et al. (2013) [95]

Mechanical	Auxotonic load	3D	mPSC, hiPSC	Calcium release, beating cells	Thavandiran et al. (2013) [23]

Electromechanical	PDMS/CNT electrodes + cyclic strain	2D	hBM-MSC	qRT-PCR	Pavesi et al. (2015) [115]
	Carbon electrodes, static stretch	3D	mPSC	Beating cells	Miklas et al. (2014) [96]
	Graphite electrodes, auxotonic contraction	3D	mPSC	Calcium release, beating cells	Godier-Furnémont et al. (2015) [97]

Coculture	Mouse endothelial cells	3D	Neonatal mouse CMs	TUNEL (apoptosis), beating cells, and connexin 43	Narmoneva et al. (2004) [108]
	Organised capillary network between artery and vein	2D	Neonatal rats CMs	Troponin T, connexin 43, and beating cells	Chiu et al. (2012) [110]
	Superior cervical ganglion neurons	3D	iPSC-CMs	Beating cells	Takeuchi et al. (2011, 2012, and 2013) [103–105], Miwa et al. (2013) [106]

**Table 2:** NICMs modelled with patient iPSC-CMs.

NICM	Culture environment	CM disease readouts^*∗*^	Selected interventions (effect)^*∗*^	Reference
Dilated cardiomyopathy	Embryoid bodies (EBs)	Disturbed sarcomeric organisation. ↑Sensitivity to positive inotropic and biomechanical stress (cyclic stretch). ↓Sarcoplasmic reticulum (SR) Ca^2+^ storage and altered Ca^2+^ handling. ↓CM contraction forces.	Overexpression of Serca2a (↑[Ca^2+^]_i_ transients and CM contraction forces). Beta-1-adrenergic blockade (resistance to biomechanical stress and improved myofilament organisation)	Sun et al. (2012) [120]

Hypertrophic cardiomyopathy	EBs	CM hypertrophy. Disturbed sarcomeric organisation. ↑Multinucleation. ↑Hypertrophy-related genes. Expression of atrial natriuretic peptide. ↑Beta-/alpha-myosin ratio. Calcineurin activation. NFATc4. ↑Myofibril content. ↑Membrane capacitance. Presence of arrhythmic waveforms. ↑Irregular beating frequencies. CM hypercontractility. Irregular Ca^2+^ transients. ↓SR Ca^2+^ release and ↑[Ca^2+^]_i_	Blockade of calcineurin-NFAT interaction (40% reduction in CM cell size). Beta-adrenergic stimulation (↑irregular Ca^2+^ transients and arrhythmia). Coadministration of beta-adrenergic blocker + beta-adrenergic agonist (↓hypertrophy, Ca^2+^ handling deficiencies, and arrhythmia). Inhibition of Ca^2+^ influx (eliminated Ca^2+^ handling abnormalities and arrhythmia, and restoration of normal beating frequency)	Lan et al. (2013) [121]

Arrhythmogenic right ventricular cardiomyopathy	EBs	↓PKP2 and plakoglobin. ↓Cx43. ↑Maximum width in CM. Disorganised Z-bands. ↑Distorted desmosomes^#^. Altered lipid droplet morphology. ↑Lipid droplets and accumulation. ↑PPAR-gamma and -alpha. ↑Susceptibility to apoptosis. ↓Beta-catenin. Abnormal [Ca^2+^]_i_ handling capability. ↓Serca2a and Na^+^/Ca^2+^ exchanger 1	Activation of canonical Wnt signalling (↓lipid droplet accumulation). Induction of adult-like energy metabolism and overactivation of PPAR-gamma signalling (increased apoptosis and lipid accumulation). Lentiviral transduction of WT PKP2 (redistribution of plakoglobin to cell membrane and nucleus, ↓apoptosis, and lipogenesis). Scavenging of reactive oxygen species (↓apoptosis). Enrichment for right ventricle-like CMs (↑lipogenesis and apoptosis)	Ma et al. (2013) [122], Caspi et al. (2013) [123], and Kim et al. (2013) [124]

Long QT syndrome 1	EBs	Longer action potentials with slower repolarisation velocities in ventricular- and atrial-like CMs. ↓K^+^ currents. Translocation of KCNQ1 from the cell surface to the endoplasmic reticulum	Transduction of WT KCNQ1 (redistribution to the cell surface of H9c2 cells). Beta-adrenergic stimulation (impaired rate adaptation of the AP and EADs), rescued by nonselective beta-blockade	Moretti et al. (2010) [74]

Long QT syndrome 2	iPSC coculture with END-2 cells [75]. EBs [125]. Directed differentiation method [126]	↑Field potential durations on microelectrode arrays. Prolonged cardiac repolarisation phase of the AP (ventricular-like CMs).↑Arrhythmogenicity. Presence of EADs. Prolonged [Ca^2+^]_i_ transients. ↓Densities of rapid delayed potassium channels. ↑Sensitivity to the antiarrhythmic drug, sotalol and the human Ether-à-go-go-related gene (hERG) blocker E-4031. ↑Sensitivity to beta-adrenoreceptor agonism	Nonspecific beta-adrenergic agonist (↑chronotropy). Pharmacological QT prolongation (arrhythmogenic behaviour). K^+^ channel enhancement (shortened APs and prevention of EADs). Ca^2+^ channel blockade (shortened APs)	Lahti et al. (2012) [76], Matsa et al. (2011) [126], and Spencer et al. (2014) [127]

Long QT syndrome 3 (Brugada syndrome)	iPSC coculture with END-2 cells	↓Inward Na^+^ current density. Defective biophysical properties of Na^+^ channel. Prolonged inactivation of Nav1.5. Mild prolongation of after-depolarising potential. Altered SR Ca^2+^ release. DADs and arrhythmias	None	Davis et al. (2012) [128] and Fatima et al. (2013) [129]

Timothy syndrome	EBs	Slower (30 bpm) and more irregular contraction rates. ↓Voltage-dependent inactivation of the L-type Ca^2+^ channel current. Longer APs (only in ventricular-like CMs). Presence of DADs. Larger and prolonged Ca^2+^ elevations	Pharmacological ↑ of voltage-dependent inactivation (↓irregular timing and amplitude of Ca^2+^ transients, and AP duration)	Yazawa et al. (2011) [130]

Catecholaminergic polymorphic ventricular tachycardia	iPSC coculture with END-2 cells	↑Ca^2+^ amplitudes and longer durations of spontaneous Ca^2+^ release events (persisted after repolarisation). DADs and arrhythmias	Catecholaminergic stimulation (↑diastolic [Ca^2+^], ↓SR Ca^2+^ content, DADs, and arrhythmias). ↑Cytosolic cAMP levels (prevented Ca^2+^ induced Ca^2+^ release events). Pharmacological stabilisation of the closed state of RyR channels (normalised Ca^2+^ spark properties)	Fatima et al. (2011) [131] and Jung et al. (2012) [132]

LEOPARD syndrome	EBs	↑Median CM surface area. ↑Sarcomere assembly. ↑NFATc4 nuclear translocation. ↑Phosphorylation of EGFR and MEK1 (in iPSCs)	None	Carvajal-Vergara et al. (2010) [133]

Hypoplastic left heart syndrome	Directed differentiation method	Less organised sarcomeres. ↓Cardiac progenitor marker Mesp1. Retarded but persistent GATA4 expression. ↑MYH6 (fast isoform). ↓cTnT and Cx43 expression. ↑Atrial natriuretic peptide. ↓Numbers and beating rates of contractile areas. Accelerated rate of Ca^2+^ transient decay. RyR and SR dysfunction. ↑Inositol trisphosphate receptor	Beta-adrenergic receptor agonism (lower increases in beat frequency)	Jiang et al. (2014) [134]

Duchenne muscular dystrophy	EBs [135]. Directed differentiation method [136, 137]. Staged: days 1–16 EBs + directed differentiation, days 20–24 maturation on hydrogel substrate with a physiological stiffness [138]	↓Dystrophin expression. Abnormal Ca^2+^ handling. ↓Numbers of spontaneously contracting embryoid bodies and cTnT-positive CMs. ↑Apoptotic markers: Ca^2+^ overload, mitochondrial damage, caspase-3 activation, and cell death	Beta-adrenergic receptor agonism (↑beating rates). Antisense oligonucleotides exon skipping (exon skipping and dystrophin expression). Minidystrophin gene construct (restored dystrophin expression). Human artificial chromosome carrying the whole dystrophin genomic locus (restored dystrophin expression including multiple isoforms and correct subcellular localisation). Oxidative stress (earlier mitochondrial permeability pore opening). Hypotonic stress (↑susceptibility). Membrane sealing (↓apoptotic markers)	Dick et al. (in press) [136], Guan et al. (2014) [138], Zatti et al. (2014) [137], and Lin et al. (2015) [139]

^*∗*^Or in other cell types where specified.

^#^Desmosomal widening correlated with lipid droplet accumulation.
